# Neutrophil Death in Myeloproliferative Neoplasms: Shedding More Light on Neutrophils as a Pathogenic Link to Chronic Inflammation

**DOI:** 10.3390/ijms23031490

**Published:** 2022-01-27

**Authors:** Dragana Marković, Irina Maslovarić, Dragoslava Djikić, Vladan P. Čokić

**Affiliations:** 1 Group for Immunology, Institute for Medical Research, National Institute of Republic of Serbia, University of Belgrade, Dr Subotića 4, POB 39, 11129 Belgrade, Serbia; irina.maslovaric@imi.bg.ac.rs; 2 Group for Molecular Oncology, Institute for Medical Research, National Institute of Republic of Serbia, University of Belgrade, Dr Subotića 4, POB 39, 11129 Belgrade, Serbia; dragoslava@imi.bg.ac.rs (D.D.); vl@imi.bg.ac.rs (V.P.Č.)

**Keywords:** myeloproliferative neoplasms, neutrophils, inflammation, cell death, apoptosis

## Abstract

Neutrophils are an essential component of the innate immune response, but their prolonged activation can lead to chronic inflammation. Consequently, neutrophil homeostasis is tightly regulated through balance between granulopoiesis and clearance of dying cells. The bone marrow is both a site of neutrophil production and the place they return to and die. Myeloproliferative neoplasms (MPN) are clonal hematopoietic disorders characterized by the mutations in three types of molecular markers, with emphasis on Janus kinase 2 gene mutation (*JAK2V617F*). The MPN bone marrow stem cell niche is a site of chronic inflammation, with commonly increased cells of myeloid lineage, including neutrophils. The MPN neutrophils are characterized by the upregulation of JAK target genes. Additionally, MPN neutrophils display malignant nature, they are in a state of activation, and with deregulated apoptotic machinery. In other words, neutrophils deserve to be placed in the midst of major events in MPN. Our crucial interest in this review is better understanding of how neutrophils die in MPN mirrored by defects in apoptosis and to what possible extent they can contribute to MPN pathophysiology. We tend to expect that reduced neutrophil apoptosis will establish a pathogenic link to chronic inflammation in MPN.

## 1. Introduction

Hematopoiesis is defined as a continuous clonal process because a single pluripotent hematopoietic stem cell can give rise to all the various cell types in the blood and lymphatic systems [1]. Commonly, hematopoiesis is polyclonal because several pluripotent hematopoietic stem cells keep perpetually producing progenitors. Instead, monoclonality is the hallmark of the myeloproliferative disorders, such that only the progeny of the transformed stem cell eventually populates the blood and marrow, which defines their malignant nature [2,3].

Philadelphia-negative (Ph-negative) myeloproliferative neoplasms (MPN) are a group of heterogeneous hematologic malignancies characterized by the clonal and unregulated proliferation of cells from several myeloid lineages: erythrocytes, leukocytes or platelets [3,4]. Ph-negative MPN neoplasms encompass three related entities, essential thrombocythemia (ET), polycythemia vera (PV) and primary myelofibrosis (PMF). Mutations in three types of molecular markers associated with Ph-negative MPN have greatly facilitated molecular diagnosis since most patients harbor acquired somatic mutation(s) in Janus kinase 2 (*JAK2*) [5,6,7,8,9], thrombopoietin receptor (*MPL*) [10,11], or calreticulin (*CALR*) genes [12,13]. *JAK2V617F* is the most common mutation detected in MPN disease with the incidence of mutation as high as ~95% in patients with PV, ~60% incidence in ET patients, and ~50% in patients with PMF [14,15]. *JAK2V617F* mutation exhibits oncogenic properties and leads to ligand-independent activation of downstream signaling pathways by constitutive phosphorylation. This in turn results in uncontrolled cell proliferation, decreased apoptosis, excessive production of reactive oxygen species and genetic instability [16,17,18,19]. The signaling pathways affected by *JAK2/MPL/CALR* mutations include the JAK-STAT (signal transducers and activators of transcription) protein families, with JAK2 and STAT5 being the key players, as well as JAK2-related pathways PI3K/AKT and Ras/MAPK [5,12,15,20,21,22]. Each type of MPN has unique as well as overlapping cell and molecular signature, all convergating towards similar signaling pathways and regulatory proteins, such as STAT, NFκB and/or HIF-1*α* [23,24,25].

The incidence of MPN is low, but the prevalence is high and comparable with lung cancer [26]. MPN neoplasms are characterized by thrombosis, progression to myelofibrosis and leukemic transformation as critical complications of the disease [27]. Most recently, the MPN neoplasms have been described as “Inflammatory Diseases” because the chronic inflammation has been described as a pivot for development and advancement of MPN from early-stage cancer to pronounced bone marrow fibrosis [26]. A frame for novel concept envisaged as “A Human Inflammation Model for Cancer Development” has been built upon combining data sources from clinical, experimental, genomic and immunological MPN studies [28].

Chronic inflammatory state in MPN is characterized by persistent activation of immune cells, elevated leukocyte and platelet counts and aberrant cytokine expression, release of various mediators, such as reactive oxygen species (ROS) and reactive nitrogen species (RNS), DNA damage, tissue destruction and myelofibrosis [28,29]. Based on current knowledge it is becoming obvious that both chronic inflammation in MPN and neoplastic transformation contribute to the pathogenesis of the disease [30]. Chronic inflammation in the bone marrow microenvironment may enhance in vivo granulocyte activation and overwhelming release of proteolytic enzymes from neutrophil granules, thereby promoting mobilization of CD34+ cells and progenitors from the bone marrow niches into the circulation (“metastasis”) [26]. Importantly, chronic inflammation in MPN may allow for the neoplastic clone to gain a selective advantage over unmutated wild type cells. Fleischman et al. 2011 [31] expose a central role of proinflammatory cytokine TNFα in promoting clonal dominance of *JAK2* mutant cells in MPN. Furthermore, Lu et al. 2015 [32] reported that inflammatory cytokine lipocalin-2 (LCN2) derived from neoplastic leukocytes contributes to the predominance of the neoplastic clone of cells in PMF and to a dysfunctional niche. In addition, NFkB is constitutively active in MPN and drives MPN-associated inflammation [25,29,33].

Another feature of MPN that has recently gained attention, due to its potential role in the pathogenesis of the disease, is the activation of intracellular multiprotein complexes known as inflammasomes which in turn mediate the inflammatory process [34,35,36]. This intracellular protein complex, expressed primarily by myeloid cells, is considered important in promoting the inflammatory response by processing highly pro-inflammatory cytokines IL-1β and IL-18 [37]. Nucleotide-binding and oligomerization domain NOD-like receptors (NLR) are a group of evolutionarily conserved intracellular pattern recognition receptors (PRRs) [38]. Among the NLRs that have been explored to operate via inflammasomes, pyrin domain containing 3 (NLRP3) inflammasome that is consisting the intracellular sensor NLRP3, the adaptor apoptosis-associated speck-like protein containing a caspase-recruitment domain (ASC), and pro-Caspase-1, has been the most studied inflammasome [37]. NLRP3 inflammasome is involved in both sterile and non-sterile inflammation [34,35]. Full NLRP3 inflammasome activation by the canonical pathway requires both a NF-κB-mediated signaling, and an activation step performed by a pathogen associated molecular patterns (PAMPs) and/or stress-associated signals released from damaged or dying cells (damage associated molecular patterns, DAMPs) [34,37]. Zhou et al. 2020 [35] showed that inflammasome-related genes were highly expressed in bone marrow cells from MPN patients and that increased expression was associated with *JAK2V617F* mutation, white blood cell counts and splenomegaly. Another inflammasome important in the context of MPN disease is the absence in melanoma 2 (AIM2), which recognizes double-stranded DNA (dsDNA) [34,36]. Sensing of dsDNA by AIM2 is crucial to mediate protection against the invading pathogens, but it also responds to dsDNA released from damaged or apoptotic host cells, resulting in the secretion of proinflammatory cytokines, thereby driving the progression of sterile inflammation [39]. Analysis conducted by Liew et al. 2016 [40] identified AIM2 as a downstream target of JAK2V617F in hematological cell lines expressing *JAK2V617F*. Induction of *JAK2V617F* leads to an inflammatory response which is consistent with the studies demonstrating the involvement of IL-1β in the development of myelofibrosis in a *JAK2V617F* mouse model [41]. In the systematic review of Găman et al. [42] it has been recently reported the existence of plethora of molecules that can act as DAMPs in MPN, such as cell-free DNA (cfDNA), microparticles and extracellular vesicles, and they may potentially activate inflammasomes and related inflammation.

In a number of studies the relationship between MPN pathophysiology and increased oxidative stress parameters has been accentuated [43,44,45,46,47]. Studies depicting the rise in both ROS production [18,21,47,48,49,50], and elevated NO-related parameters [47], implicated oxidative and nitrosative impact [47] on MPN pathogenesis. ROS and RNS act as modulators of protein and lipid kinases and phosphatases, membrane receptors, ion channels, transcription factors [51]. ROS augmentation might inhibit phosphatase activities leading to a prolonged phosphorylation-dependent JAK/STAT signaling [21]. The *JAK2V617F* mutation induces the accumulation of ROS in the hematopoietic stem cell compartment, where overproduction of ROS act as a mediator of oxidative stress and genomic instability [21,28,29].

In MPN, in vivo activated leukocytes, platelets, megakaryocytes, and bone marrow stromal cells continually release diverse inflammatory mediators, including inflammatory cytokines, chemokines, reactive oxygen species (ROS) and reactive nitrogen species (RNS) [21,28]. However, a malignant clone, which can involve cells from various myeloid lineages, has been implicated in maintaining MPN inflammation as a key player [18,24,25]. Disruption of hematopoiesis and the establishment of an increased production of clonal myeloid cells gives rise to one of three classical MPN phenotypic outcomes: PV is characterized by an absolute increase in red cell mass, and increased number of leukocytes and platelets (“trilineage growth”), ET is characterized by overproduction of platelets and PMF with deregulation of the megakaryocyte and granulocyte lineages [3,45,52]. It is worth notifying that cells from all myeloid lineages in MPN neoplasms, including neutrophils, may belong to a malignant clone regardless of their total number in the circulation in a particular MPN phenotype.

Despite the fact that in response to cancer neutrophils can interact with many parts of the host immune system in up-down regulation of cancer progression, there is no article to describe how neutrophils and neutrophil clonality (mutant allele burden) can orchestrate the evolution of a pathological microenvironment in MPN disease. In this review article we present our perspective, after having compiled all the relevant data on the role of neutrophils in MPN, focusing this time on one aspect: the role of neutrophil death in MPN mirrored by defects in apoptosis.

Apoptosis of neutrophils supervises the duration and the intensity of an inflammatory response, and the extent of neutrophil-mediated tissue damage [53]. The following facts are in support of this statement: (1) neutrophils possess distinct types of cargo in neutrophil granules and secretory vesicles, such as myeloperoxidase (MPO), matrix metalloproteinase 9 (MMP9), proteinase 3 (PR3), cathepsin G, neutrophil gelatinase-associated lipocalin (NGAL), neutrophil elastase (NE), [54,55,56]; (2) neutrophils are not only translationally active cells but are transcriptionally active cells as well and they express inflammatory mediators such as cytokines and chemokines [57,58]; (3) they produce two major precursors of all reactive oxygen and nitrogen species - superoxide and NO [51]; (4) human neutrophils have compromised DNA repair system [59,60,61]; (5) neutrophils influence multiple aspects of hematopoietic niche physiology and regulate hematopoietic stem cells (HSC) and hematopoietic progenitor cells (HPC) quiescence and proliferation [62,63].

The dysregulation of apoptosis in MPN is a general phenomenon, due to constitutive activation of JAK2/STAT, PI3K/AKT and Ras-MAPK/ERK signaling pathways, that modulate the expression of proteins involved in apoptosis [19,64,65]. A great attention was paid to the dysregulated expression of megakaryocyte apoptosis and their involvement in MPN pathogenesis [19,66,67,68]. In this review we place our focus of attention on neutrophils. A number of inflammatory cytokines, including cytokines such as G-CSF and GM-CSF, that can signal through JAK/STAT pathways, can prolong neutrophil survival [57,69,70]. In humans, neutrophils are the most prevalent type of all circulating leukocytes [71,72] and the most abundant myeloid population in the bone marrow [63], even in MPN [73]. Megakaryocytes are large but rare cells in human bone marrow [73,74] and despite the significant megakaryocyte hyperplasia in MPN, they remain a small percentage of cells in the bone marrow [73].

Therefore, we believe that neutrophils in MPN are not receiving attention they deserve as cells unique and specific to the inflammatory response, and with undeniable potential to gain malignant nature. Instead they could be placed in the midst of major events in MPN: (1) MPN disease is characterized by upregulated JAK2 tyrosine kinase signaling, secondary to acquired HSC) mutations, that promotes myeloproliferation and mediates resistance to apoptosis [19]; (2) the MPN bone marrow stem cell niche is a site of chronic inflammation, with commonly increased cells of myeloid lineage, including neutrophils [3,28]; (3) MPN neutrophils display malignant nature, they are in a state of activation, and with deregulated apoptotic machinery [3,75,76,77,78]; (4) reduced neutrophil apoptosis supports unwanted excessive inflammation [72,79,80]; (5) chronic inflammation has been described as a pivot for development and advancement of MPN from early-stage cancer to pronounced bone marrow fibrosis [26]; (6) apoptosis-modulating neutrophil drugs represent a potential strategy for MPN treatment [81]. Therefore, our crucial interest in this review is better understanding of the role of neutrophil death in MPN mirrored by defects in apoptosis, and to what possible extent they can contribute to MPN pathophysiology.

## 2. Neutrophils

Traditionally, neutrophils were seen as terminally differentiated cells destined to commit suicide on their one-way mission from bone marrow to the tissue. Our current knowledge related to the novel neutrophil functional competencies, which describes neutrophils as cells capable of acquiring functionally important phenotypic features that most likely reflect the differential inflammatory stimulus, have broadened our understanding of their diverse roles including their role as predictors of disease severity, in human health and disease [63,79,80]. Neutrophils are an essential component of the innate immune response, but they are also a major contributor to inflammation. While performing their fundamental role, fighting against pathogens, neutrophils can exert under pathological conditions, such as inflammatory diseases, potentially toxic activity to tissues, via the production and release of diverse mediators, such as ROS, RNS [51], cytokines (proinflammatory IL-1*β*, IL-6, IL-12, TNFα, monocyte chemotactic protein (MCP)-1, lipocalin 2, oncostatin M and anti-inflammatory IL-1ra, TGF-*β*), chemokines (IL-8, growth-related oncogene-*α* (GRO-*α*), MIP-1α and *β*, macrophage inflammatory protein Mip-3 α/β, IP-10, MIG, I-TAC, Mip-3) [57,82,83,84], proteases [85], adhesion molecules [80,86] and neutrophil extracellular trap (NET) components, such as cfDNA, mitochondrial DNA, extracellular histones and granule proteins [87,88,89]. Human neutrophils express key components of the NLRP3 and AIM2 inflammasomes machinery and process and release proinflammatory cytokines IL-1*β* and IL-18 [90]. Important to note, due to their large number compared to other leukocytes, neutrophils are a relevant source of proinflammatory cytokines [37,82,91].

Consequently, neutrophil homeostasis is tightly regulated through balance between granulopoiesis, bone marrow storage and release, intravascular margination, and clearance of dying cells [63,92]. The proper elimination of neutrophils is important to prevent overexuberant immune response to ongoing inflammation, cytotoxic tissue damage, initiation, or progression of autoimmune processes [72,79] or tumorigenesis [80].

Apparent fascination by neutrophils, ever since their discovery in the late 19th century, has led in the last decade or two of intense study of neutrophil physiology, to more comprehensible body of knowledge of their longevity and destiny from the moment they would egress the bone marrow. Neutrophils play prominent immune roles, but they are not just infantry of the innate immune system destined to attack and destroy invading organisms, they modulate the inflammatory response during infection and repair the tissue damage through their active interaction with the host immune system. Beyond all this a striking and unanticipated discovery of non-immune properties of neutrophils, supporting homeostasis as well as complex diseases with an inflammatory component such as cancer, has emerged recently.

### MPN Neutrophils

In MPN, the number of neutrophils is increased [3], they are in a state of activation [75,76] while their apoptosis is deregulated [77,78]. MPN neutrophils are both clonal and polyclonal, and the rate of clonal dominance varies among MPN phenotypes [3,24]. When they are clonal, MPN neutrophils express different level of *JAK2V617F* allele burden [3,24,47,76,93]. However, regardless of the *JAK2* mutational status, MPN-associated neutrophils are characterized by the upregulation of JAK-STAT target genes [22]. The neutrophil *JAK2V617F* allele burden can over-estimate the burden at the progenitor cell level [94]. Moreover, activated JAK2 signature is present in granulocytes of MPN patients with somatic *CALR* mutations [22].

Granulocytes in MPN are characterized by increased level of intracellular ROS production [47,49], which initiates oxidative stress that incurs damage to macromolecules [47], both intracellular and to the neighboring cells. They are source of proinflammatory cytokines in MPN [24,32,95,96,97], with an impact on chronic proinflammatory state that contributes to disease pathogenesis. They are involved in tromboinflammation and thrombosis manifestation [98,99,100] as well as in fibrosis [32,101] in MPN. Diverse cytokine receptors expressed on the neutrophil cell surface, that transduce signals through the JAK/STAT pathways and affect their crucial functions [102], represent the most obvious link between neutrophils and MPN pathophysiology.

## 3. Neutrophil Cell Death

In humans, neutrophils are the most prevalent type of all circulating leukocytes [71,72] and the most abundant myeloid population in the bone marrow [63]. They have been traditionally considered as short-lived immune cells with circulating half-life of 8–20 h in humans, although their life expectancy extends up to 4 days after migration into tissue [103], which imposes demands for their constant production and release from the bone marrow into blood [63]. This instructs a basal production rate of 5 × 10^10^ to 1 × 10^11^ neutrophils per day [71,92]. The number of neutrophils is increased in infections, inflammatory diseases, and some leukemias/cancers [80,104].

As neutrophils are constantly generated in the bone marrow this implies that large number of neutrophils must be eliminated every day [105,106]. Neutrophil cell death is context-dependent, and can include several processes of programed cell death, such as apoptosis, NETotic death, autophagy-associated death, pyroptosis, oncosis, and necroptosis, as well as process of passive cell death such as necrosis [72,107,108,109,110,111]. However, apoptosis is the most common physiological cell death of neutrophils [53] that is also involved in the pathogenesis of several diseases including cancer, degenerative diseases of the central nervous system and immune system dysfunction [112].

### 3.1. Apoptosis

Apoptosis represents a conserved mechanism of programmed cell death [106]. Neutrophils die by constitutive apoptosis, an essential mechanism for neutrophil functional shutdown regulation [53,106]. Apoptotic neutrophils are characterised by a series of typical morphological features such as cytoplasmic shrinkage, nuclear condensation, DNA fragmentation and membrane blebbing [113]. They must display engulfment signals, known as “find-me” and “eat me” signals on their plasma membranes, such as the expression of today most studied phosphatidylserine receptor or calreticulin [70,72,113], which permit recognition and phagocytic engulfment of dying cells by macrophages (efferocytosis) [70]. Apoptosis depends on the balance between pro- (e.g., BAX, BAK) and anti-apoptotic (e.g., BCL-2, BCL-B, BCL-xL, BCL-W, MCL-1, A1) factors, which are both members of the B-cell lymphoma-2 (BCL-2) family of proteins [114]. The anti-apoptotic protein myeloid cell leukemia sequence 1 (MCL-1) plays a key role in the regulation of neutrophil apoptosis [113,115,116]. Important to note, cell cycle regulatory proteins, that in other cell types serve to control proliferation, in neutrophils, surprisingly, regulate apoptosis and survival [106,117]. Thus, as neutrophil-specific regulatory factors, proliferating cell nuclear antigen (PCNA), myeloid nuclear differentiation antigen (MNDA) and cyclin-dependent kinases (CDKs) are identified [106,117]. These neutrophil-specific regulatory proteins are localized in the nucleus of proliferating cells, while in mature neutrophils they are located in the cytoplasm where they act as pro-survival (PCNA, CDKs) or pro-apoptotic (MNDA) factors [106,117]. Neutrophil apoptosis is usually initiated by the intrinsic or extrinsic apoptotic pathway [118]. The intrinsic pathway mediates constitutive neutrophil apoptosis, which is driven by the permeabilisation of the mitochondrial outer membrane and subsequent release of cytohrome c from mitochondria, which leads to the activation of caspase-9, which in turn causes the activation of frequently activated death protease, caspase-3 [70,119]. The extrinsic pathway of neutrophil apoptosis is induced by ligation of TNF receptor superfamily cell surface death receptors, which drives caspase-8 dependent activation of caspase-3 [70,113].

**Figure 1:**
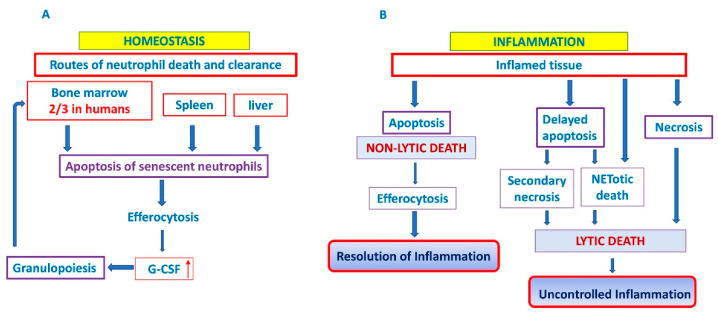
Neutrophil cell death and clearance. In homeostatic conditions, clearance of aged neutrophils is believed to occur via their uptake into the bone marrow (predominantly in humans), liver and spleen. It generates homeostatic signals leading to granulocyte colony-stimulating factor (G-SCF) release that drives granulopoiesis as a positive feedback loop (**A**). In inflammatory conditions, two outcomes are possible: efferocytosis of apoptotic neutrophils and resolution of inflammation or delayed apoptosis and progression towards lytic cell death and uncontrolled inflammation (**B**). G-CSF: granylocyte colony stimulating factor. ↑ denotes increased expression.

Neutrophil apoptosis represents a pro-resolution mechanism that limits the extent of inflammation and consequently tissue injury, since efferocytosis occurs before the plasma membrane of apoptotic cells becomes leaky [113]. Therefore, whereas nonlytic apoptotic cell death allows controlled removal of cells and promote healing and anti-inflammatory responses, pathways ending with loss of cell membrane integrity, such as NETotic death, necrosis and necroptosis, results in leakage of pro-inflammatory and toxic cell contents into the extracellular space and may elicit deleterious proinflammatory responses [110,111] (Figure 1B). More importantly, dysregulated apoptotic neutrophil death, either upregulated or downregulated, is often linked solely to chronic inflammatory diseases like cancer [111] or autoimmune diseases [114,120]. Reduced neutrophil apoptosis supports inflammation, stroma remodeling, tumor angiogenesis and metastasis [111,120].

### 3.2. Clearance of Apoptotic Neutrophils

In the absence of an infectious challenge, homeostatic clearance of aged neutrophils is believed to occur via their uptake into the liver, spleen and bone marrow, where they are phagocytosed by tissue-resident macrophages and dendritic cells (DC) [92,117,121]. In a steady state, about a third of all circulating senescent neutrophils in mice are phagocytosed in the bone marrow resident macrophages [122], while the level of phagocytosed neutrophils in the human bone marrow can go as high as 67% (two-thirds) of circulating neutrophils [121]. Thus, bone marrow is the place of ultimate neutrophil production [123], and a site of their homeostatic clearance [121,124,125] (Figure 1A).

Clearance of peripheral blood senescent neutrophils, that express chemokine receptor CXCR4 and home back to the bone marrow [62,126] generates homeostatic signals leading to granulocyte colony-stimulating factor (G-SCF) release that drives granulopoiesis as a positive feedback loop for homeostatic regulation of circulating neutrophils [62,91]. This process is closely regulated in both mice and humans [62,126]. A mechanism that regulates neutrophil production and completes feedback loop responsible for the tight control of neutrophil number in the peripheral tissue and in the circulation was demonstrated in in vitro and complementary in vivo studies by Stark et al. [62], and involves the IL-23/IL-17/G-CSF feedback circuit. Phagocytosis of apoptotic neutrophils by tissue dendritic cells and macrophages reduces the production of IL-23, which in turn reduces the production of IL-17, leading to less G-CSF and reduced proliferation, and differentiation of neutrophil precursors in the bone marrow [62]. Conversely, when neutrophil migration into tissue is blocked by adhesion molecules deficiency, the result of this event is decreased phagocytosis, increased IL-23, IL-17 and G-CSF, and the production of neutrophils is enhanced [62,69,91].

In the case of tissue injury and/or infection neutrophils follow the gradient of chemoattractants, such as CXCL8 (IL-8) that determines the direction of neutrophil migration into the tissues [111,117]. After fulfilling their biological function (elimination of pathogens by phagocytosis, degranulation, release of ROS or formation of NETs, etc.), neutrophils are removed in situ by macrophages and by dendritic cells [72]. A portion of neutrophils migrates back to the vasculature, then they reverse migrate to marrow, passing through to the lungs en route to the bone marrow, where they undergo apoptosis [127]. Phagocytosis of apoptotic neutrophils by macrophages is not just dead cell disposal system because tissue phagocytes provide a mechanism for the safe disposal of apoptotic material. Signaling pathways will regulate the phagocytic response and determine whether apoptotic cell clearance is immunologically “silent” or even anti-inflammatory [128]. It suppresses the production of G-CSF to limit the inflammation [62,69] and reduces the number of viable and activated neutrophils without releasing the potentially harmful enzymes and ROS, thereby facilitating the resolution of inflammatory response. Furthermore, efferocytosis has an important influence on the resolution of inflammation, through secretion of anti-inflammatory cytokines, such as TGF-β, IL-10 and VEGF, by macrophages [129,130]. On the other hand, an elevated level of TGF-β could drive unrestrained fibrotic responses, like in MPN [72,131]. Neutrophil longevity during inflammation may be extended by various stimuli, including pattern recognition signals, growth factors (G-CSF, GM-CSF) or chemokines [69,70,72]. As mentioned above, delayed death and clearance of neutrophils could progress either to uncontrolled inflammation of related tissue [132] or toward lytic cell death, such as secondary necrosis [70,72,113,133], or NETotic death [134], which usually triggers inflammation in both cases [120,133]. Thus, it has been shown that macrophages produce the proinflammatory cytokines and chemokines, such as TNF-α and IL-8, largely due to proteases liberated by lysed neutrophils [135].

## 4. The Interplay between Bone Marrow and Neutrophils

Neutrophils spend most of their lifetime in the bone marrow: during granulopoiesis [123], in a reserve pool in both humans and mice [136], in marginated pool [121] and for clearance/death [121]. Neutrophils are the most abundant leukocytes. Accordingly, as much as 55% to 60% of the bone marrow in humans is dedicated to neutrophil production [104]. Mature neutrophils that are not released immediately from the bone marrow are referred to as the bone marrow reserve [137]. The process of mature neutrophil release from the bone marrow under homeostasis is not constant, instead it fluctuates according to the time of the day with distinct diurnal peak in mobilization in humans [55]. The bone marrow reserve of mature neutrophils in humans is estimated to be 6 × 10^11^ cells [138], approximately 20 times the number of neutrophils in circulation [71] (Table 1). The egress of mature neutrophils from the bone marrow into the periphery depends on a balance between chemokines and their receptors: the CXCR4/CXCL12 axis mediates the retention of neutrophils within the bone marrow [126,136,139], whereas CXCL1/CXCL2 signaling through CXCR2 receptor promotes their release [140,141]. Efficient mobilization of neutrophils requires G-CSF, which stimulates the expression of CXCR2 ligands on the bone marrow endothelial cells [141]. Chemokine CXCL12, the major ligand for CXCR4, is produced constitutively by the bone marrow stromal cells under homeostatic conditions in mice and in humans [136]. Under steady-state conditions, CXCR4/CXCL12 axis is controlled circadianally by the autonomic nervous system, i.e., sympathetic signaling [142]. As neutrophils age in the systemic circulation, they express an increased number of surface CXCR4 [126,143], followed by decreased expression of CXCR2 [143]. This surface marker profile of senescent neutrophils ensures homing back to the bone marrow where aged neutrophils are destroyed by bone marrow macrophages [141]. The level of phagocytosed neutrophils in the human bone marrow can go as high as two-thirds of circulating neutrophils [121].

In the blood, neutrophils are divided in two compartments: the circulating pool and the marginated pool [144]. The main neutrophil marginated pools are in the liver, spleen, and bone marrow [144]. It is estimated that the bone marrow pools 25% of blood neutrophils in humans [121].

The production, circulation, margination and clearance of neutrophils is altered by inflammatory stimuli, such as those encountered in acute and chronic inflammatory conditions [144,145]. The data obtaned by Szczepura et al. [121] have shown that for patients with chronic obstructive pulmonary disease (COPD), the proportions of neutrophils pooling and undergoing destruction in the bone marrow appear to be increased. It should be noted that COPD patients have dysregulated, reduced, neutrophil apoptosis [146]. MPN bone marrow stem cell niche is a site of chronic inflammation, characterized by increased myeloproliferation, deregulated neutrophil apoptosis and, presumably, disbalanced neutrophil marginal and reserve pool, altered by inflammatory stimuli, with impact on resolution of inflammation. The question is to what kind of problems the disturbed neutrophil pool balance might lead to in the organs where the marginated cells are found, especially under inflammatory conditions [147]. This aspect in MPN has not been considered so far. We believe that disruption of these compartments in MPN can have a major impact on the resolution of inflammation in MPN, in the bone marrow and in the circulation, thereby affecting the pathogenesis and progression of the disease. Also, the data on neutrophil efferocytosis in human bone marrow in malignancies, especially hematological, are limited. Arnold and Kahwash [148] in their work described bone marrow macrophages containing neutrophils or neutrophil nuclear fragments, significantly associated with malignancy (when compared to bone marrow biopsies for nonneoplastic conditions), in particular Hodgkin lymphoma, which they called “fragmentophages”. Hodgkin lymphoma is known to be associated with increased production of cytokines, such as IL-6 and IL-10 [149], VEGF, IL-8, bFGF as well as LDH activity [150], and myeloid hyperplasia in the bone marrow [148], the conditions similar to those seen in MPN.

On the other hand, neutrophils are important regulators of hematopoietic niche, and consequently of HSC and HPC activity [63]. Neutrophils can induce local inhibition of niches and favor the release of HSC/HPC from the bone marrow [143]. Conversely, they can limit HSC/HPC release from the bone marrow and prevent excessive HSC mobilization [151]. In addition, they can control hematopoietic niche from distant, extramedullary tissues [62,152]. More importantly, neutrophils regulate HSC/HPC quiescence and proliferation [62,63]. What has become obvious lately is the fact that neutrophils influence multiple aspects of physiological niche, from maintenance of the mesenchymal niche to HSC quiescence, which calls for prompt assessment of their contribution to inflammatory diseases and hematological malignancies [63].

## 5. Apoptosis of MPN Neutrophils

Little is known about how neutrophils die in MPN. As stated above, even under homeostatic conditions large numbers of neutrophils must be eliminated every day. In MPN, the number of neutrophils is increased due to myeloproliferation and prolonged survival, they are in a state of activation, and with deregulated apoptotic machinery [3,75,76,77,78]. The question is how they die and where the clearance takes place?

Andersen et al. [153] used mathematical modeling as a proof of concept that chronic inflammation may be a driving force for clonal evolution and MPN disease progression. Their integrated inflammation-MPN model tend to couple cell dynamics to a basal inflammatory response, as seen in common infections, where the amount of dead cells provokes the immune response and stimulates the renewal of stem cells. The delineated mathematical modeling has pinpointed the inflammation as being a highly potent stimulus for clonal evolution and myeloproliferative neoplasm progression. In addition, they placed bone marrow macrophages and their significance in the midst of all other cells, both with regard to inflammatory cytokines release and also with regard to the development of myelofibrosis. However, this approach [153] predicted only the role of neutrophils as phagocytes capable of eliminating dead cells. This modeling should be further validated by predicting the role of neutrophils as cells specific to inflammatory response, either as cells that harbour driver mutations defining MPN or as polyclonal population of cells, both populations acting synergistically in pro-inflammatory environment. The key question is: what is happening with neutrophil cell death and its clearance in MPN neoplasms, because the number of neutrophils is elevated, they are displaying clonal nature, indicating clonal population within the neutrophil population, while the neutrophil apoptosis is dysregulated. The bone marrow, otherwise the site of neutrophil production [123] and clearance of normal aged neutrophils [121], in MPN becomes a site of sustained chronic inflammation [28], with immune pathophysiology most likely in the myeloid part of immune response [24,154]. At some point, bone marrow can start harbouring a clonal population of myeloid/neutrophil cells with tendency to turn, over prolonged period of time, benign immune pathophysiology into malignant MPN clonal disorder [3,26].

The dysregulation of apoptosis in MPN is a general phenomenon, due to constitutive activation of JAK2/STAT, PI3K/AKT and Ras-MAPK/ERK signaling pathways, given that STAT transcription factors modulate the expression of proteins involved in apoptosis, such as c-Myc, cyclin D, MCL-1 and BCL-XL [19,64,65]. Activation of PI3K/AKT pathway leads to BCL-XL up-regulation and inhibition of megakaryocyte apoptosis while ERK phosphorilation activates BAD (apoptosis activator), and BCL-2 (apoptosis inhibitor), the overall result is diminished apoptosis in MPN [68].

Tognon et al. [78], Mambet et al. [19] as well as Diaconu et al. [155] provided an overview of dysregulation process of programmed cell death by apoptosis in Ph-MPN. The literature they presented showed the importance of both intrinsic and extrinsic apoptotic pathways in the pathogenesis of MPN. Dysregulation of apoptosis related genes that may contribute to MPN physiopathology has been observed so far for erythroid cells [156], megakaryocytes [66,67,68] bone marrow CD34+ hematopoietic stem cells [157,158] and peripheral blood leukocytes [157,158]. However, a great attention was paid to the dysregulated expression of megakaryocyte apoptosis and their involvement in MPN pathogenesis [19,66,67,68].

In this review we place our focus of attention on neutrophils. A number of inflammatory cytokines, including cytokines such as G-CSF and GM-CSF, that can signal through JAK/STAT pathways, can prolong neutrophil survival [57,69,70]. GM-CSF induced activation of STAT3 signaling, in cooperation with PI3K, has been implicated in Mcl-1 upregulation and delayed apoptosis in normal human neutrophils [159]. In regard to MPN, Mesa et al. [77] have examined neutrophil apoptotic resistance in PMF. The highest resistance to apoptosis they observed in PMF neutrophils with the highest mutant allele burden in contrast to the lowest resistance observed in wild-type *JAK2*. Tognon et al. [160] reported deregulation of the extrinsic apoptotic pathway in the leukocytes of patients with MPN: *FAS*, *C-FLIP* and *TRAIL* levels were increased in PV, anti-apoptotic gene *FAIM* was increased in PMF, while *DR5* expression was decreased in ET patients (Table 2). Further, Tognon et al. [157] investigated the involvement of the intrinsic apoptotic pathway in the pathophysiology of patients with ET and PMF in both bone marrow CD34+ hematopoietic cells and peripheral blood leukocytes. In the MPN pathophysiology, they reported dysregulation of the intrinsic pathway in apoptosis activation, namely, they observed increased expression of anti-apoptotic genes *A1*, *BCL-2*, *BCL-X_L_*, and *BCL-W* in leukocytes of ET and PMF patients, and decreased expression of pro-apoptotic *BID* and *BIM_EL_* genes in leukocytes of ET patients (Table 2).

A study characterizing the gene expression profile of granulocytes, isolated from PV patients, observed upregulation of protease inhibitors that act on the proteases involved in the promotion of neutrophil apoptosis, as well as upregulation of several antiapoptotic and survival factors (e.g., p38 MAPK) [161]. Granulocytes isolated from PV patients were found to express an increased amount of heat shock protein 70 (HSP70), which counteracts caspase-dependent apoptosis, i.e., reduces caspase activation by preventing BAX translocation to mitochondria [162]. In neutrophils isolated from ET patients, Hui et al. [163] revealed differently expressed proteins involved in apoptotic and inflammatory pathways. Čokić et al. [164] performed an extensive proteomic and gene expression analysis of granulocytes from MPN patients. They found notable up-regulation of regulatory signaling molecules responsible for myeloid cell apoptosis, such as RAC2 (proteomic studies), S100A8/9, coronin 1A (CORO1A) (detected by both proteomic and microarray analysis), and coiled-coil domain containing 88A protein coding gene (*CCDC88A*), detected by microarray analysis. In PMF patients they demonstrated overexpression of *PYCARD*, which encodes bipartite protein that consists of a PYrin domain and a CAspase Recruitment Domain and is a key mediator in apoptosis and inflammation (Table 2). Socoro-Yuste et al. [50] found that calreticulin (CALR), known to be involved in calcium homeostasis and apoptotic signaling (“eat me” signal), was overexpressed in *JAK2V617F* compared with wild-type *JAK2* granulocytes. To summarize, observed dysregulation of neutrophil apoptosis in MPN was expressed as an up-regulation of both anti-apoptotic (*C-FLIP*, *CORO1A*) and pro-apoptotic (*S100*, *PYCARD*, *FAS*, *TRAIL*) factors, as well as down-regulation of pro-apoptotic (*BID*, *BIM*, *DR5*) factors (Table 2).

Epigenetic control of gene expression by hypermethylation may silence the gene expression, and in the case of gene-specific promoter methylation of apoptosis-related (pro-apoptotic) genes, it can induce cell resistance to death. By analyzing the methylation profile of apoptosis-related genes in leukocytes of MPN patients, Tognon et al. [165] reported that pro-apoptotic genes, including *TP53* gene, might be deregulated by epigenetic mechanisms.

## 6. Chronic Inflammation in MPN Due to Dysregulated Neutrophil Cell Death

Neutrophil prolonged survival and activity, due to its dysregulated apoptosis, participate in the disease pathogenesis by supporting inflammation, stroma remodeling, tumor angiogenesis and metastasis [111]. Neutrophils may propagate chronic inflammation via elevated levels of inflammatory cytokines [57,82,83,84], ROS, RNS [51], or NET components release [87,88,89] and by recruiting and activating further leukocytes, including neutrophils [80,111,166,167] (Table 3). Even excessive apoptosis or when cell debris is improperly cleared, may result in the release of inflammatory mediators and contribute to chronic inflammation [120].

**Figure 2:**
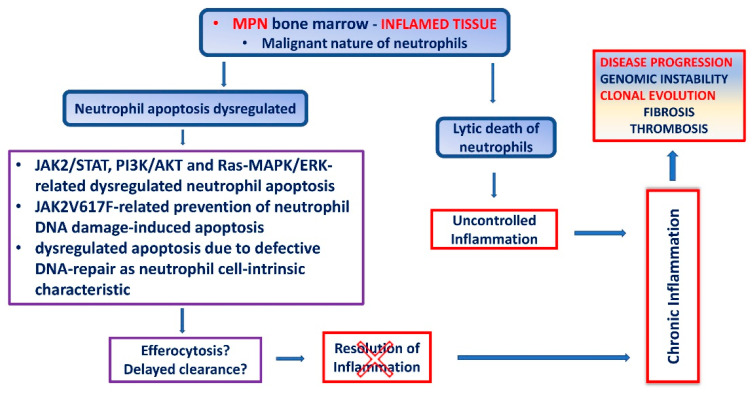
Putative role of dysregulated neutrophil cell death in MPN. Neutrophil prolonged survival and activity, due to its dysregulated apoptosis, supports inflammation and regulates eventual outcome of a disease (sustained genetic instability, positive selection of neoplastic clone, consequent dissease progression). MPN: myeloproliferative neoplasms; JAK: Janus kinase 2; STAT: signal transducers and activators of transcription; PI3K: phosphatydilinositol-3-kinase; AKT: RAC-alpha/beta serine/threonine-protein kinase/protein kinase B; MAPK: mitogen activated protein kinase; ERK: extracellular signal-regulated kinase.

Dysregulated MPN neutrophil apoptosis and their prolonged survival supports chronic inflammation. Chronic inflammation has been described as a pivot for the development and advancement of MPN from early-stage cancer to pronounced bone marrow fibrosis [17,26,28,30,32,154,168,169,170,171]. At this point we can argue that dysregulated neutrophoil apoptosis may fundamentally contribute to the pathogenesis of MPN disease (Figure 2). Moreover, delay of neutrophil cell death and clearance drives them towards lytic death, such as secondary necrosis [70,72,113,133], or NETotic death [134], which usually triggers inflammation in both cases (Figure 1B) [120,133]. Even though it has become obvious for secondary necrosis or other forms of lytic death of neutrophils to take place in MPN, no reliable data are available. When relying on the NET studies in MPN we came across the data presented in the context of thrombosis, and not in the context of lytic NETotic death and inflammation in MPN pathology [172,173,174,175]. One phenomenon regarding lytic neutrophils death was observed in cell-in cell contact with megakaryocytes in PMF, i.e., pathological emperipolesis of neutrophils within megakaryocytes [101,176]. In this mutual destructive action megakaryocytes cause the destruction of the neutrophils and release of their lysosomes into megakaryocyte cytoplasm, which in turn causes the lysis of megakaryocytes, leading to destruction of megakaryocyte storage organelles and leakage of α–granular contents, such as growth factors PDGF and TGF-β, into the bone marrow microenvironment, and to the generation of myelofibrosis [176].

## 7. Impaired Neutrophil Apoptosis in MPN as a Consequence of Defective DNA Repair Mechanisms

Maintenance of genomic stability relies, among other processes, on the coordinated action of DNA replication, DNA repair and cell-cycle regulation [178]. Following DNA damage, cells undergo adequate DNA repair mechanisms, combined with the removal by apoptosis of damaged cells with irreparable genomic lesions [178].

As opposed to other cell types in the hematopoietic system that posess efficient DNA-repair mechanism [61,179], human neutrophils have compromised DNA repair system [59,60,61]. Macrophages, DC [61] and eosinophils [60] express/re-express the relevant DNA repair proteins, while in the case of monocytes the data are contradictory: Salati et al. [60] reported that monocytes are DNA-repair competent, as oppose to Ponath et al. [61] who pointed out to impaired DNA-repair in monocytes. As neutrophils and monocytes arise from the same precursor, myeloblast, Ponath et al. [61] drew attention to the importance of determining whether the downregulation of DNA repair occurs at the myeloid progenitor or precursor stage, since the outcome in mitotically active cells would be different from that of mature, post-mitotic, cells.

Endogenously generated ROS induces base damage and single-strand breaks (SSB) as a primary event, and potentially lethal double-strand breaks (DSB) as a secondary [180]. Although neutrophils are type of cells that generate large quantities of ROS, with potential to induce endogenous DNA damage, human neutrophils lack the capacity to repair both double and single strand DNA breaks [59,60,61]. Sallmyr et al. [59] have shown reduced expression of DNA-dependent protein kinase (DNA-PK) in neutrophils, indicating downregulation of nonhomologous end-joining (NHEJ) repair pathway of oxidative damage. The data with regard to the cellular repair capacity of neutrophils presented by Ponath et al. [61] indicate strong downregulation of DNA repair and DNA damage response factors. The key proteins required for efficient DNA SSB repair, i.e., XRCC1, PARP-1 and ligase III, as well as for DNA DSB repair, i.e., ATM, ATR and DNA-PKCS in neutrophils were not expressed. Also, neutrophils, despite accumulation of DSB, did not show γ-H2AX foci formation [61].

The DNA repair is an energy–consuming process and it seems that genetic stability is not necessary for primary functions of neutrophils, especially in view of the fact that they are mature, non-dividing, short-lived cells, that undergo constitutive apoptosis [61].

Neutrophils in MPN are both clonal and polyclonal, and the rate of clonal dominance varies among MPN phenotypes [3,24]. It is however expected for neutrophils to accumulate somatic mutations over time, and alongside the prolonged survival due to impaired apoptosis, gain a selection advantage and lead the way in its environment towards malignant nature of MPN. When they are clonal, MPN neutrophils express different level of *JAK2V617F* allele burden [3,24,47,76,93]. The neutrophil *JAK2V617F* allele burden can over-estimate the burden at the progenitor cell level [94]. It has been shown that JAK2V617F expression is associated with increased DNA instability and DNA-damage in MPN pathophysiology [169,181]. Furthermore, although normal polyclonal neutrophils per se are the cells that generate the large quantities of ROS, with potential to induce endogenous DNA damage, granulocytes in MPN are characterized by elevated level of intracellular ROS production, which initiates oxidative stress that incurs damage to macromolecules [47,49].

In addition to defective DNA-repair in neutrophils, as their cell-intrinsic characteristic, there is a report that argues JAK2V617F and BCR-ABL kinases to be of relevance for inhibition of DNA damage-induced apoptosis, in granulocytes from MPN patients, a process likely to drive disease evolution [168]. Upon DNA damage, activation of plasma membrane transporter amiloride-sensitive sodium-hydrogen exchanger isoform 1 (Na+/H+ exchanger isoform 1, NHE-1) leads to deamidation of the antiapoptotic protein BCL-xL, which reduces the ability of BCL-xL to sequester and inhibit the BCL-2 homology 3 (BH3)-only family of proapoptotic proteins, thereby promoting apoptosis [182]. In their study, Zhao et al. [168] found that this signaling pathway, leading from DNA damage to deamidation of BCL-xL and consequent apoptosis, is inhibited in granulocytes from PV and CML patients. This observation raises a possibility that aberrant JAK2V617F and BCR-ABL kinases may prevent the pro-apoptotic response and increase the accumulation of DNA damage within the mutant clone, with final impact on disease progression [168,169]. In mature neutrophils, post-mitotic and pro-inflammatory cells, it can be assumed that reduced apoptosis and delayed death and clearance would support inflammation, which creates the conditions for positive selection of neoplastic clone, sustained genetic instability and disease progression.

Therefore, genomic instability in MPN neutrophils and/or their precursor and progenitor cells, can evolve as a direct consequence of: (1) constitutive JAK2-mediated signaling [169]; (2) JAK2V617F kinase related prevention of DNA damage-induced apoptosis by inappropriate regulation of cell survival pathways [168]; (3) defective DNA-repair as their cell-intrinsic characteristic [59,60,61]. Regardless of the cause of genomic instability in MPN neutrophils, apoptosis is delayed/dysregulated, and with final impact on positive selection of neoplastic clone and disease progression (Figure 2).

## 8. Targeting Neutrophil Apoptosis in MPN Therapy

Despite recent advances in MPN treatment, the search for novel therapeutic approaches and novel cell and molecular targets in MPNs continues. The ability to modulate the life or death of a cell carries great therapeutic potential. The mechanisms for neutrophil recruitment are strictly context-related and highly dynamic, thus they rely on the specific type of disease and the associated cell types [147]. Lately, the emerging concept of neutrophil heterogeneity and plasticity in both basal and inflammatory conditions, although many neutrophil subsets that are described in the literature might only represent neutrophils in different activation and maturation states, has proposed a therapeutic potential by specific targeting of neutrophil subsets in pathological conditions. However the ongoing challenge how to target detrimental, while promoting protective, neutrophil phenotypes remains. Furthermore, during an infection the shift of neutrophils from the marginating pool into the circulating pool happens quickly, which is why neutrophils might also be mobilized from the marginated sites, and this could mean an additional therapeutic potential [92]. The question is to what kind of problems the disturbed neutrophil pool balance might lead to in the organs where the marginated cells are found, especially under inflammatory conditions [147]. Even though neutrophil modulation carries a risk, therapeutic options to modify neutrophil phenotypes from harmful to beneficial are needed, and “depletion of neutrophils is not an option” [138]. Driving neutrophil apoptosis in terms of overcoming resistance to apoptosis is a potential concomitant therapeutic strategy for MPN treatment, given the fact that neutrophil apoptosis supervises the duration and the intensity of an inflammatory response. Promising pro-apoptotic therapies under current clinical trials include combination of JAK1/2 related and BCL-xL related inhibitors [183]. Modulation of the NHE-1/Bcl-xL deamidation signaling pathway can be another therapeutic approach to overcome resistance to apoptosis in MPN [168]. Further, pro-apoptotic drugs may directly inhibit anti-apoptotic proteins such as MCL-1 and A1. Although presently we do not have any proof to refer to of possible overexpression of MCL-1 in MPN neutrophils, it has been shown that GM-CSF induced activation of STAT3 signaling, in cooperation with PI3K, is implicated in Mcl-1 upregulation and delayed apoptosis in normal human neutrophils [159]. In addition, Mcl-1 plays a key role in the regulation of neutrophil apoptosis [113,115,116]. Clinical evaluation of MCL-1 inhibitors for hematological malignancies is currently underway, with a major clinical objective to determine a safe therapeutic window for this class of inhibitors, in accordance with physiological roles of Mcl-1 [184]. In addition to emerged novel approaches to therapeutic targeting of neutrophil granulocytes, neutrophils can be used as carriers of therapeutics, as well. In this setting, the attention is focused on neutrophil-specific survival regulatory factors (PCNA, MNDA, CDKs). CDK inhibitor R-roscovitine, that can overcome the effects of pro-survival stimuli such as GM-CSF, TNFα, and LPS, is of particular interest [106,185,186]. In the study of Robertson et al. [81] it is shown that polymersomes, nanometer-sized synthetic vesicles, can effectively encapsulate and deliver CDK inhibitor R-roscovitine into human neutrophils, to promote neutrophil apoptosis in vitro and help resolve a model of neutrophilic inflammation in vivo, without evoking detrimental effects on neutrophil viability or IL-8 release. Furthermore, Chu et al. [187] reviewed two types of drug delivery systems based on neutrophils: neutrophils as carriers and neutrophil-membrane-derived nanovesicles and their potential applications in treating inflammation and cancers. Bisso et al. [188] investigated nanomaterial interactions with human neutrophils with implications for design of intravenously delivered particulate formulations targeting circulating neutrophils. The importance of these studies is the opportunity to deliver therapeutics to bone marrow or distal sites of inflammation, which is of special interest in MPN treatment. To summarize, we are looking forward to future innovations in the area of neutrophil characterization and its use in the management of inflammatory diseases.

## 9. Conclusions

The intention of this review was to provide better insight and understanding of the role of neutrophil death in MPN, and how and to what possible extent they can contribute to MPN pathophysiology, in view of dysregulated apoptotic machinery. Chronic inflammation has been described as a major driving force for the development and advancement of MPN. We tend to expect that reduced neutrophil apoptosis, which favors excessive inflammation, would pave the way for stroma remodeling, fibrosis, thrombosis, tumor angiogenesis and metastasis, and, above all, it would promote positive selection of neoplastic clone and sustain genetic instability. The other aspect of ongoing events is the interplay between bone marrow and neutrophils, and the fact that bone marrow becomes inflammatory microenvironment in MPN.

## Figures and Tables

**Table 1:** The bone marrow neutrophil compartments in humans.

Human Bone Marrow		
	Homeostasis	MPN
**Granulopoiesis**	55% to 60% of the bone marrow is dedicated to neutrophil production [104]	Increased myeloproliferation, increased neutrophil number [3,28]
**Neutrophil death and clearance**	Up to two-thirds of circulating neutrophils [121]	Deregulated [77,78]
**Neutrophil marginal pool**	25% of total blood pool [121]	Disbalanced?
**Neutrophil reserve pool**	6 × 10^11^ cells [138]; contains ~20 times the number of neutrophils in circulation [71]	Disbalanced?

MPN: myeloproliferative neoplasms.

**Table 2:** Expression of apoptosis-related factors in granulocytes of MPN patients.

Pro-Apoptotic	MPN	Anti-Apoptotic	MPN
*BID* ↓, BID ↓	ET [157]	*C-FLIP* ↑	PV [160]
*BIM* ↓	ET [157]	CORO1A ↑	MPN [164]
*BAD* ↑	ET, PMF [157]	*FAIM* ↑	PMF [160]
*DR5* ↓	ET [160]	*A1* ↑	ET, PMF [157]
*PYCARD* ↑	PMF [164]	*BCL-2* ↑	ET, PMF [157]
*FAS* ↑	PV [160]	*BCL-W* ↑	ET, PMF [157]
*TRAIL* ↑	PV [160]	*BCL-XL* ↑	ET, PMF [157]
		BCL-XL ↑	PMF [157]
S100 ↑	MPN [164]	RAC2 ↑	MPN [164]
*TP53* methylation	ET, PMF [165]		

MPN: myeloproliferative neoplasms; ET: essential thrombocythemia; PV: polycythemia vera; PMF: primary myelofibrosis; BID: BH3-interacting domain death agonist; BIM: BCL-2-interacting mediator of cell death; BAD: Bcl-2-associated death promoter; DR: death receptor; PYCARD: Pyrin domain and a Caspase Recruitment Domain; TRAIL: TNF-related apoptosis inducing ligand; C-FLIP: FLICE-like inhibitory protein; CORO1A: coronin 1A; FAIM: FAS apoptosis inhibitory molecule; BCL: B-cell lymphoma. ↑ denotes increased expression; ↓ denotes decreased expression. Gene expression: written in italics; protein expression: written in normal font/not italicized.

**Table 3:** Neutrophil pro-inflammatory capacity in MPN.

Neutrophil Pro-Inflammatory Capacity		MPN
**Cytokine production**	proinflammatory IL-1β, IL-6, IL-12, TNFα, MCP-1, lipocalin 2, oncostatin M and anti-inflammatory IL-1ra, TGF-β [57,82,84]	Increased [24,32,96,97]
**Chemokine production**	IL-8, GRO-α, MIP-1α and β, Mip-3 α/β, IP-10, MIG, I-TAC, Mip-3 [82,83]	Increased [24]
**ROS and RNS production**	Superoxide, H_2_O_2_, NO [51]	Increased [28,47,49]
**NET components release**	cfDNA, mitochondrial DNA, extracellular histones, granule proteins [87,88,89]	Increased [172,173,174,175]
**NLRP3 and AIM2 inflammasome**	process and release proinflammatory cytokines IL-1β and IL-18 [90]	Increased expression [35]
**Neutrophil granules and secretory vesicles content**	MPO, MMP9, proteinase 3, cathepsin G, neutrophil gelatinase-associated lipocalin-2, neutrophil elastase [54,55,56,177]	Increased [32,85,96,97,174]

MPN: myeloproliferative neoplasms; ROS: reactive oxygen species; RNS: reactive nitrogen species; NET: neutrophil extracellular trap; NLRP3: NOD-like receptor pyrin domain containing 3; AIM2: absence in melanoma 2; cfDNA: cell-free DNA; MPO: myeloperoxidase; MMP9: matrix metalloproteinase 9.

## Data Availability

Not applicable.

## Abbreviations

AIM2	absence in melanoma 2
BCL-2	B-cell lymphoma-2
CALR	calreticulin
CDKs	cyclin-dependent kinases
cfDNA	cell-free DNA
COPD	chronic obstructive pulmonary disease
DAMPs	damage associated molecular patterns
DC	dendritic cells
DSB	double-strand breaks
dsDNA	double-stranded DNA
ET	essential thrombocythemia
HPC	hematopoietic progenitor cell
HSC	hematopoietic stem cell
JAK2	Janus kinase 2
LCN2	lipocalin-2
MAPK	mitogen activated protein kinase
MCL-1	myeloid cell leukemia sequence 1
MMP9	matrix metalloproteinase 9
MNDA	myeloid nuclear differentiation antigen
MPL	myeloproliferative leukemia virus, thrombopoietin receptor
MPN	myeloproliferative neoplasms
MPO	myeloperoxidase
NE	neutrophil elastase
NET	neutrophil extracellular trap
NGAL	neutrophil gelatinase-associated lipocalin
NLRP3	NOD-like receptor pyrin domain containing 3
NLRs	NOD-like receptors
PAMPs	pathogen associated molecular patterns
PCNA	proliferating cell nuclear antigen
PI3k	phosphatydilinositol-3-kinase
PMF	primary myelofibrosis
PRRs	pattern recognition receptors
PV	polycythemia vera
RNS	reactive nitrogen species
ROS	reactive oxygen species
SSB	single-strand breaks
STAT	signal transducers and activators of transcription
